# Polymorphisms within Autophagy-Related Genes Influence the Risk of Developing Colorectal Cancer: A Meta-Analysis of Four Large Cohorts

**DOI:** 10.3390/cancers13061258

**Published:** 2021-03-12

**Authors:** Juan Sainz, Francisco José García-Verdejo, Manuel Martínez-Bueno, Abhishek Kumar, José Manuel Sánchez-Maldonado, Anna Díez-Villanueva, Ludmila Vodičková, Veronika Vymetálková, Vicente Martin Sánchez, Miguel Inacio Da Silva Filho, Belém Sampaio-Marques, Stefanie Brezina, Katja Butterbach, Rob ter Horst, Michael Hoffmeister, Paula Ludovico, Manuel Jurado, Yang Li, Pedro Sánchez-Rovira, Mihai G. Netea, Andrea Gsur, Pavel Vodička, Víctor Moreno, Kari Hemminki, Hermann Brenner, Jenny Chang-Claude, Asta Försti

**Affiliations:** 1 Genomic Oncology Area, GENYO, Centre for Genomics and Oncological Research Pfizer/University of Granada/Andalusian Regional Government, 18016 Granada, Spain; josemanuel.sanchez@genyo.es (J.M.S.-M.); manuel.jurado.sspa@juntadeandalucia.es (M.J.); 2 Hematology Department, Virgen de las Nieves University Hospital, 18012 Granada, Spain; 3 Instituto de Investigación Biosanataria IBs.Granada, 18012 Granada, Spain; 4 Department of Medicine, University of Granada, 18016 Granada, Spain; 5 Department of Medical Oncology, Complejo Hospitalario de Jaén, 23007 Jaén, Spain; francisco.garcia.verdejo.sspa@juntadeandalucia.es (F.J.G.-V.); oncopsr@yahoo.es (P.S.-R.); 6 Area of Genomic Medicine, GENYO, Centre for Genomics and Oncological Research Pfizer/University of Granada/Andalusian Regional Government, 18016 Granada, Spain; manuel.martinez@genyo.es; 7 Division of Molecular Genetic Epidemiology, German Cancer Research Center (DKFZ), 69120 Heidelberg, Germany; abhishek@ibioinformatics.org (A.K.); m.daSilvaFilho@dkfz.de (M.I.D.S.F.); k.hemminki@dkfz-heidelberg.de (K.H.); 8 Institute of Bioinformatics, International Technology Park, Bangalore, Karnataka 560066, India; 9 Manipal Academy of Higher Education (MAHE), Manipal, Karnataka 576104, India; 10 Catalan Institute of Oncology, Bellvitge Biomedical Research Institute (IDIBELL), Consortium for Biomedical Research in Epidemiology and Public Health (CIBERESP) and University of Barcelona, 08908 Barcelona, Spain; adiez@iconcologia.net (A.D.-V.); v.moreno@iconcologia.net (V.M.); 11 Department of Molecular Biology of Cancer, Institute of Experimental Medicine, Academy of Sciences of the Czech Republic, 142 00 Prague, Czech Republic; ludmila.vodickova@iem.cas.cz (L.V.); veronika.vymetalkova@iem.cas.cz (V.V.); pavel.vodicka@iem.cas.cz (P.V.); 12 Institute of Biology and Medical Genetics, 1st Medical Faculty, Charles University, 12800 Prague, Czech Republic; 13 Biomedical Centre, Faculty of Medicine in Pilsen, Charles University in Prague, 323 00 Pilsen, Czech Republic; 14 Consortium for Biomedical Research in Epidemiology and Public Health (CIBERESP), 28029 Madrid, Spain; vicente.martin@unileon.es; 15 Instituto de Biomedicina (IBIOMED), Universidad de León, 24071 León, Spain; 16 Life and Health Sciences Research Institute (ICVS), School of Medicine, University of Minho, 4710-057 Braga, Portugal; mbmarques@med.uminho.pt (B.S.-M.); pludovico@med.uminho.pt (P.L.); 17 ICVS/3B’s—PT Government Associate Laboratory, Braga, Guimarães, Portugal; 18 Department of Medicine I, Institute of Cancer Research, Medical University of Vienna, Borschkegasse 8a, A-1090 Vienna, Austria; stefanie.brezina@meduniwien.ac.at (S.B.); andrea.gsur@meduniwien.ac.at (A.G.); 19 Network Aging Research (NAR), University of Heidelberg, 69115 Heidelberg, Germany; k.butterbach@dkfz.de (K.B.); h.brenner@dkfz-heidelberg.de (H.B.); 20 Department of Internal Medicine and Radboud Center for Infectious Diseases, Radboud University Nijmegen Medical Center, 6525 GA Nijmegen, The Netherlands; RterHorst@cemm.oeaw.ac.at (R.t.H.); Yang.Li@helmholtz-hzi.de (Y.L.); mihai.netea@radboudumc.nl (M.G.N.); 21 Division of Clinical Epidemiology and Aging Research, German Cancer Research Center (DKFZ), Im Neuenheimer Feld 280, 69120 Heidelberg, Germany; m.hoffmeister@dkfz.de; 22 Centre for Individualised Infection Medicine (CiiM) & TWINCORE, Joint Ventures between the Helmholtz-Centre for Infection Research (HZI) and the Hannover Medical School (MHH), 30625 Hannover, Germany; 23 Department for Immunology & Metabolism, Life and Medical Sciences Institute (LIMES), University of Bonn, 53115 Bonn, Germany; 24 Division of Cancer Epidemiology, German Cancer Research Center (DKFZ), Im Neuenheimer Feld 280, 69120 Heidelberg, Germany; 25 Faculty of Medicine and Biomedical Center in Pilsen, Charles University in Prague, 30605 Pilsen, Czech Republic; 26 Division of Preventive Oncology, German Cancer Research Center (DKFZ), National Center for Tumour Diseases (NCT), 69120 Heidelberg, Germany; 27 German Cancer Consortium (DKTK), German Cancer Research Center (DKFZ), 69120 Heidelberg, Germany; 28 Division of Cancer Epidemiology, German Cancer Research Center (DKFZ), 69120 Heidelberg, Germany; j.chang-claude@dkfz.de; 29 Genetic Tumour Epidemiology Group, University Medical Center Hamburg-Eppendorf, University Cancer Center, 20246 Hamburg, Germany; 30 Division of Pediatric Neurooncology, German Cancer Research Center (DKFZ), German Cancer Consortium (DKTK), 69120 Heidelberg, Germany; 31 Hopp Children’s Cancer Center (KiTZ), 69120 Heidelberg, Germany

**Keywords:** colorectal cancer, autophagy, genetic variants, susceptibility

## Abstract

**Simple Summary:**

We investigated the influence of autophagy-related variants in modulating colorectal cancer (CRC) risk through a meta-analysis of genome-wide association study (GWAS) data from four large European cohorts. We found that genetic variants within the *DAPK2* and *ATG5* loci were associated with CRC risk. This study also shed some light onto the functional mechanisms behind the observed associations and demonstrated the impact of *DAPK2*_rs11631973_ and *ATG5*_rs546456_ polymorphisms on the modulation of host immune responses, blood derived-cell counts and serum inflammatory protein levels, which might be involved in promoting cancer development. No effect of the *DAPK2* and *ATG5* polymorphisms on the autophagy flux was observed.

**Abstract:**

The role of genetic variation in autophagy-related genes in modulating autophagy and cancer is poorly understood. Here, we comprehensively investigated the association of autophagy-related variants with colorectal cancer (CRC) risk and provide new insights about the molecular mechanisms underlying the associations. After meta-analysis of the genome-wide association study (GWAS) data from four independent European cohorts (8006 CRC cases and 7070 controls), two loci, *DAPK2* (*p* = 2.19 × 10^−5^) and *ATG5* (*p* = 6.28 × 10^−4^) were associated with the risk of CRC. Mechanistically, the *DAPK2*_rs11631973G_ allele was associated with IL1 β levels after the stimulation of peripheral blood mononuclear cells (PBMCs) with *Staphylococcus aureus* (*p* = 0.002), CD24 + CD38 + CD27 + IgM + B cell levels in blood (*p* = 0.0038) and serum levels of en-RAGE (*p* = 0.0068). *ATG5*_rs546456T_ allele was associated with TNF α and IL1 β levels after the stimulation of PBMCs with LPS (*p* = 0.0088 and *p* = 0.0076, respectively), CD14+CD16− cell levels in blood (*p* = 0.0068) and serum levels of CCL19 and cortisol (*p* = 0.0052 and *p* = 0.0074, respectively). Interestingly, no association with autophagy flux was observed. These results suggested an effect of the *DAPK2* and *ATG5* loci in the pathogenesis of CRC, likely through the modulation of host immune responses.

## 1. Introduction

Colorectal cancer (CRC) is the third most common cancer in developed countries and the second leading cause of morbidity and mortality in both men and women worldwide [1]. Despite modern advances in the diagnosis, surgery, and treatment of CRC, approximately 40% of patients die because of the disease [2]. Although it is well established that genetic events contribute to CRC pathogenesis [3] and that the combination of these factors with the gut microbiome, diet, environmental or even epigenetic factors provides an unprecedented opportunity to improve CRC diagnosis, disease stratification and the tailoring of treatments [4], the precise molecular mechanisms that lead to CRC development and its progression remain elusive.

Increasing evidence suggests that autophagy, a cellular catabolic degradation pathway, is a central process driving colorectal tumorigenesis and cytotoxic response to chemotherapeutic agents [5]. It has been demonstrated that hypoxic cancer cells use autophagy as a way to obtain additional nutrients and energy for cell survival and expansion [6]. Autophagy has been reported to be deregulated in CRC [7], and autophagy molecules such as Beclin 1, p62/sequestosome and LC3 are overexpressed in a high percentage of colorectal carcinomas [7]. In addition, genetic studies have shown that autophagy-related genes are frequently mutated in colon cancer cells and that positive regulators of autophagy (such as *Bif-1*) are implicated in the development of various cancers, including colon adenocarcinoma [8]. Autophagy could act as a treatment resistance mechanism prolonging tumour cell survival and it also contributes to the enrichment and survival of CRC stem cells under oxaliplatin treatment [9]. In support of the role of autophagy in modulating response to treatment, the administration of hydroxychloroquine (HCQ), an autophagy inhibitor, was found to enhance anti-cancer activity of the histone deacetylase inhibitor, vorinostat (VOR), in preclinical models and early phase clinical studies of metastatic CRC [10]. On the other hand, uncontrolled autophagy has been reported to limit inflammation and modulate multicellular immunity processes (affecting macrophages, T and B cells, neutrophils, and dendritic cells) and memory responses, but also cell differentiation, genomic stability and even leads to cell death through different pathways [11]. In this regard, it has been reported that autophagy controls immunity through NLRP3 inflammasome-dependent signals but also through ATG proteins that act independently on the inflammasome [12,13,14]. Despite the findings that point towards an important role of autophagy in CRC development, tumour cell survival and host immunity, the role of this biological process in CRC is not fully understood and might depend on how it is regulated during the course of the disease [11].

Studies on genetic variants in autophagy-related genes and their association with CRC risk may lead to further insight into mechanisms. So far, only a limited number of autophagy-related single nucleotide polymorphisms (SNPs) have been reported to be associated with CRC risk [15,16] and patient survival [17]. Therefore, we aimed to comprehensively evaluate germline variants within autophagy-related genes in relation to CRC risk using four large European cohorts. In addition, because autophagy has been linked to host immunity [17,18], we assessed the functional consequences of the SNPs that showed associations with CRC risk by conducting in vitro stimulatory experiments in a large cohort of healthy donors as well as through the analysis of a large panel of serum inflammatory biomarkers and steroid hormones and the comprehensive characterisation of blood-derived immune cell populations and the autophagy flux status.

## 2. Material and Methods

### 2.1. Study Populations

This study included 4 large European populations. The discovery study sample included 7998 subjects (4485 CRC patients and 3513 controls) ascertained through the DACHS study conducted in southwest Germany. Demographic and clinical characteristics of recruited CRC patients and healthy controls are shown in Table S1. Briefly, CRC cases were recruited from patients who received in-patient treatment in a hospital of the Rhein–Neckar–Odenwald region due to a first diagnosis of CRC. Controls were frequency-matched according to gender, 5-year age groups, and county of residence, and were then contacted by mail and follow-up calls. Demographic information as well as information on colonoscopies, diet, anthropometry, physical activity, medication (including statins, nonsteroidal anti-inflammatory drugs (NSAIDs), menopausal HRT), reproductive factors, lifestyle factors, and family history was collected during a face-to-face interview by trained interviewers using a standardised questionnaire. To be eligible, participants had to be at least 30 years old and capable of completing the interview. The three other study samples included the CRCGen study consisting of 948 Spanish CRC patients and 1076 healthy controls (Table S2), the COloRectal cancer Study of Austria (CORSA) that included 968 cases and 848 colonoscopy-negative controls, and the Czech Republic Colorectal Cancer Study (CCS) that recruited 1605 CRC cases and 1633 healthy controls. Written informed consent from all study subjects was collected and all the studies were approved by the ethical review committees of the participating institutions (Medizinische Fakultät Heidelberg, DACHS, Darmkrebs: Chancen der Verhütung durch Screening 310/2001; HUB, PR151/14; the Medical University of Vienna, MUW, EK Nr. 703/2010, the “Ethikkommission Burgenland”, KRAGES, 33/2010; and the Institute of Experimental Medicine in Prague, Czech Republic). Information about these four populations has been described in detail elsewhere [3,19,20,21,22].

### 2.2. Gene and SNP Selection, Association Analysis, and Meta-Analysis

**Figure 1:**
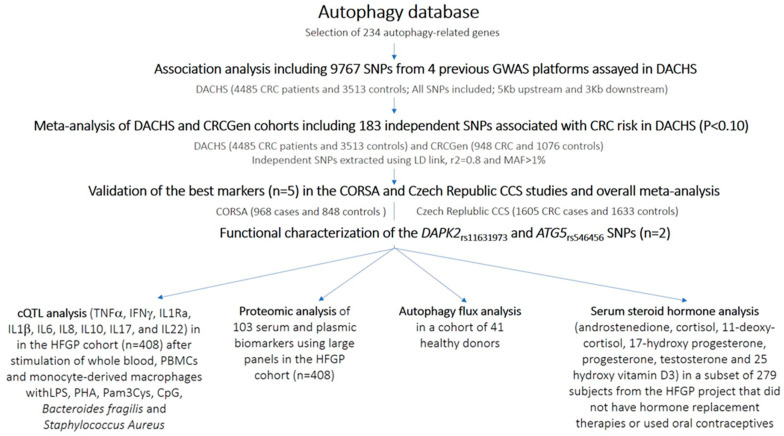
Flow diagram of the study.

A total of 234 autophagy-related genes were selected on the basis of their presence in the autophagy database (http://autophagy.lu/index.html, accessed on 13 December 2019; Table S3) and association estimates for all genotyped or imputed SNPs within or near these genes (5 Kb upstream and 3 Kb downstream) were extracted from 4 genome-wide association studies (GWAS) for CRC conducted in the DACHS population between 2003 and 2016. Details about the genotyping platforms used and the number of CRC cases and controls analysed in each study are shown in Table S4. Genotyping, quality control filtering, and imputation protocols used in these studies have been described in detail elsewhere [23,24,25,26]. Altogether, 9767 SNPs in the autophagy-related genes, either genotyped or imputed, were available from GWAS in the DACHS sample. We performed an overall logistic regression analysis adjusted for 3 principal component analyses (PCAs) and identified 925 SNPs showing an association with CRC risk at *p* < 0.10. Of those, 183 SNPs were considered independent SNPs according to the information provided by LD link pairwise linkage disequilibrium *r*^2^ < 0.8 (https://ldlink.nci.nih.gov/?tab=home, accessed on 13 December 2019). Using GWAS data from the CRCGen study [3], we conducted a meta-analysis of the DACHS and CRCGen populations for the 183 independent SNPs and used the *I*^2^ statistic to assess statistical heterogeneity between the studies. The pooled odds ratio (OR) was computed using the fixed-effect model. The multiple testing significance threshold was set to 0.00027 (0.05/183 independent SNPs) to the meta-analysis results. After the meta-analysis, the most interesting associations (*p* < 0.002) were further validated using GWAS data from the CORSA (948 CRC cases and 1076 controls) and Czech Republic CCS (1605 CRC cases and 1633 controls) studies. A workflow diagram of this study is shown in Figure 1.

### 2.3. Genotyping of Imputed SNPs in the CORSA and Czech Republic CCS Cohorts

To validate the genotypes of imputed SNPs that showed the lowest *p*-value in the association analysis (*NRG3*_rs11196336_, *DAPK2*_rs11635284_, *EGFR*_rs2075108_, *TP73*_rs4648553_ and *ATG5*_rs546456_), genotyping of the whole CORSA population and a subset of the Czech CCS study (1031 CRC cases and 886 controls) was carried out at GENYO (Centre for Genomics and Oncological Research, PTS Granada, Granada, Spain) using KASPar^TM^ genotyping technology (LGC Genomics, Hoddesdon, UK) or Taqman^®^ SNP Genotyping assays (Thermo Fisher Scientific, Foster City, CA, USA) according to previously reported protocols [27]. For internal quality control, ~5% of samples were randomly selected and included as duplicates. Concordance between the imputed and the genotyped samples for the SNPs analysed was ≥99.5%.

### 2.4. Functional Association of the Autophagy-Related Variants with Immune Responses

In order to determine the functional role of the most interesting SNPs after the meta-analysis of the 4 cohorts (independent SNPs showing a *p*-value lower than 0.001), we conducted cytokine stimulation experiments in the 500 Functional Genomics cohort from the Human Functional Genomics Project (HFGP; http://www.humanfunctionalgenomics.org/site/, accessed on 13 December 2019), an excellent cohort to determine the influence of genomic variation on the variability of immune responses. The HFGP study was approved by the Arnhem-Nijmegen Ethical Committee (no. 42561.091.12) and biological specimens were collected after informed consent was obtained. We investigated whether any of the SNPs associated with CRC in the meta-analysis of all study populations significantly correlated with levels of 9 pro- and anti-inflammatory cytokines (TNF α, IFN γ, IL1Ra, IL1 β, IL6, IL8, IL10, IL17, and IL22) after the stimulation of whole blood, peripheral blood mononuclear cells (PBMCs) or monocyte-derived macrophages (MDM) from 408 healthy subjects with LPS (1 or 100 ng/mL, Sigma-Aldrich, St. Louis, MO, USA), PHA (10 µg/mL, Sigma, St. Louis, MO, USA), Pam3Cys (10 µg/mL, EMC microcollections, Tübingen, Germany), or CpG (100 ng/mL, InvivoGen, San Diego, CA, USA), but also common bacterial components of the human intestinal microbiota (*Bacteroides fragilis* and *Staphylococcus aureus* representing Gram-negative and Gram-positive bacteria, respectively). After log transformation, linear regression analyses adjusted for age and sex were used to determine the correlation of the SNPs with cytokine expression quantitative trait loci (cQTLs). All analyses were performed using R software (http://www.r-project.org/, accessed on 13 December 2019) using custom scripts in the R programming language based on existing functions such as lm (stats). In order to account for multiple comparisons, we used a significance threshold of 0.00046 (0.05/2 independent SNPs within *DAPK2* and *ATG5* loci × 9 cytokines × 6 stimulants).

Detailed protocols for PBMCs isolation, macrophage differentiation and stimulation assays have been reported elsewhere [28]. Briefly, PBMCs were washed twice in saline and suspended in medium (RPMI 1640) supplemented with gentamicin (10 mg/mL), L-glutamine (10 mM) and pyruvate (10 mM). PBMC stimulations were performed with 5 × 10^5^ cells/well in round-bottom 96-well plates (Greiner Bio-one, Frickenhausen, Germany) for 24 h in the presence of 10% human pool serum at 37 °C and 5% CO_2_. Supernatants were collected and stored in −20 °C until used for ELISA. LPS (100 ng/mL), PHA (10 µg/mL) and Pam3Cys (10 µg/mL), CpG (100 ng/mL), *Bacteroides fragilis* (NCTC 10584) and *Staphylococcus aureus* (ATCC 25923) were used as stimulators for 24 or 48 h. *Bacteroides fragilis* and *Staphylococcus aureus* were heat-killed for 30 min at 95 °C and 100 °C, respectively. Whole blood stimulation experiments were conducted using 100 μL of heparin blood that was added to a 48-well plate and subsequently stimulated with 400 µL of LPS, PHA (final volume 500 µL) and *Staphylococcus aureus* for 48 h at 37 °C and 5% CO_2_. Supernatants were collected and stored in −20 °C until used for ELISA. Concentrations of human TNF α, IFN γ, IL1Ra, IL1 β, IL6, IL8, IL10, IL17, and IL22 were determined using specific commercial ELISA kits (PeliKine Compact, Amsterdam, or R&D Systems), in accordance with the manufacturers’ instructions. When values were below or above the detection limit of the ELISA, the corresponding limit was used.

### 2.5. Correlation between Autophagy-Related SNPs and Serum Steroid Hormone Levels

Next, we investigated the correlation of the most interesting SNPs with levels of 7 serum steroid hormones (androstenedione, cortisol, 11-deoxy-cortisol, 17-hydroxy progesterone, progesterone, testosterone and 25 hydroxy vitamin D3) in 279 subjects selected from the HFGP project that did not have hormone replacement therapies or used oral contraceptives. Serum steroid hormone levels were determined by chromatography–tandem mass spectrometry after protein precipitation and solid-phase extraction following previously reported protocols [29]. After log transformation, correlation between steroid hormone levels and autophagy-related SNPs was evaluated by linear regression analysis adjusted for age and sex. The significance threshold was set to 0.0036 considering the number of independent SNPs tested (*n* = 2) and the number of hormones determined (*n* = 7).

### 2.6. Correlation of Autophagy SNPs and Blood Cell Counts and Serum/Plasmatic Proteomic Profile

We also investigated the effect of autophagy variants on cell-level variation by using a set of 91 manually annotated immune cell populations and genotype data from the HFGP cohort that included 408 healthy subjects (Table S5). Cell populations were measured by 10-color flow cytometry (Navios flow cytometer, Beckman Coulter, Miami, FL, USA) after blood sampling (2–3 h), and cell count analysis was performed using Kaluza software (Beckman Coulter, v.1.3). In order to reduce inter-experimental noise and increase statistical power, cell count analysis was performed by calculating parental and grandparental percentages, which were defined as the percentage of a certain cell type within the subpopulation of the cells from which it was isolated [30]. Detailed laboratory protocols for cell isolation, reagents, gating, and flow cytometry analysis have been reported elsewhere [29] and the accession number for the raw flow cytometry data and analysed data files are available upon request to the authors (http://hfgp.bbmri.nl, accessed on 13 December 2019). A proteomic analysis was also performed in serum and plasma samples from the HFGP cohort. Circulating proteins were measured using the commercial Olink^®^ Inflammation panel (Olink, Sweden) that resulted in the measurement of 103 different biomarkers (Table S6). Protein levels were expressed on a log2-scale as normalised protein expression values and normalised using bridging samples to correct for batch variation. Considering the number of proteins (*n* = 103) and polymorphisms (*n* = 2) tested, a *p*-value of 0.00024 was set as the significance threshold for the proteomic analysis.

### 2.7. Impact of Autophagy-Related Variants on the Autophagy Flux

In order to accurately determine the role of autophagy SNPs in modulating autophagy, we investigated their impact on the autophagy flux in a cohort of 41 European healthy donors. For that purpose, we isolated peripheral blood mononuclear cells (PBMCs) from whole blood by density gradient centrifugation using Histopaque^®^, and we treated them for 2 h with 10 µM of bafilomycin A1 or 10 mM of metformin to inhibit or induce autophagy, respectively. A total of 5 × 10^−5^ PBMCs were plated in each well for stimulatory and inhibitory experiments and treated with metformin or bafilomycin A1 alone or in combination. Untreated cells were used as experimental controls. After treatment, cells were harvested and protein extraction was performed with 50 µL of lysis buffer (1% NP-40, 500 mM Tris HCL, 2.5 M NaCl, 20 mM EDTA, phosphatase and protease inhibitors—from Roche—at pH 7.2). Twenty (20) µg of the total protein were resolved in a 12% SDS gel and transferred to a Nitrocellulose membrane for 10 min in a Trans-Blot Turbo transfer system. Membranes were then blocked for 1 h using Tris buffered saline (TBS) with 0.1% Tween 20 (TBST) containing 5% BSA and incubated overnight at 4 °C with the polyclonal primary antibodies at 1:1000 in 1% BSA (Rabbit anti-LC3A/B Antibody, Cell-Signaling and Mouse anti-Actin antibody, Merck Millipore, Darmstadt, Germany). After washing with tris-buffered saline-tween (TBS-T), nitrocellulose membranes were incubated with the corresponding secondary antibodies (IgG anti-Rabbit for LC3A/B and IgG anti-Mouse for Actin). Protein levels were detected after incubation with SuperSignal West Femto Maximum Sensitivity Substrate (Thermofisher) or Clarity Western ECL Substrate (Bio-Rad, Hercules, CA, USA). Digital images of the Western blots were obtained in a ChemiDoc XRS System (Bio-Rad) with Quantity One software V4.6.5 (Bio-Rad). Autophagy flux was determined as the difference in the LC3-II/Actin ratio between cells treated or not with bafilomycin A1 and/or metformin, and lineal regression analyses adjusted for age and sex were used to determine the correlation between autophagy-related SNPs and autophagy flux values. A significance threshold of 0.0125 was set according to the quotient of 0.05 and the number of SNPs tested (*n* = 2) and the treatments administrated in vitro (*n* = 2).

### 2.8. In Silico Functional and eQTL Analysis

Finally, we analysed whether selected SNPs could have a functional effect in a wide variety of human cell types using data from publicly available bioinformatic tools such as Haploreg (http://www.broadinstitute.org/mammals/haploreg/haploreg.php, accessed on 13 December 2019) [31], ENCODE annotation data (https://genome.ucsc.edu/ENCODE/, accessed on 13 December 2019), Regulome (www.regulome.org, accessed on 13 December 2019) and GTex portal (https://gtexportal.org/home/, accessed on 13 December 2019), Blood eQTL browser (https://genenetwork.nl/bloodeqtlbrowser/, accessed on 13 December 2019). 

## 3. Results

This comprehensive association study included a total of 15,076 subjects (8006 CRC cases and 7070 controls) (Figure 1). In the DACHS sample (4485 CRC patients and 3513 controls), association analysis of 9767 genotyped and imputed SNPs in 234 autophagy-related genes yielded 183 independent SNPs (*r*^2^ < 0.8) that were associated at *p* < 0.10. These SNPs were selected for the first meta-analysis of the DACHS and CRCGen samples, comprising 5433 CRC cases and 4589 controls. The meta-analysis revealed the most significant associations with risk of CRC for a single SNP within the NRG3 gene and an LD block including 14 variants in the DAPK2 locus (*r*^2^ > 0.90; Table 1 and Table S7).

Each copy of the *NRG3*_rs11196336_ C allele increased the risk of developing CRC by 17% (*p* = 1.85 × 10^−5^), whereas in the *DAPK2* locus three SNPs in strong LD increased the risk by 15% (rs11633496, rs11633611 and rs11631973; *p* = 7.71 × 10^−5^–7.99 × 10^−5^; Table 1 and Table S7). Additionally, we found potentially interesting associations for SNPs within the *EGFR, TP73* and *ATG5* loci. Considering these results, we decided to advance the *NRG3* and *DAPK2* SNPs for replication, due to showing the most significant associations with CRC risk, but also those that were associated with CRC risk at *p* < 0.002 (21 SNPs representing five independent signals after excluding LOC100128105, which was predicted to be a hypothetical protein by the Guide to the Human Genome (www.cshlp.org/ghg5_db/recinfo/87/8750.shtml, accessed on 13 December 2019)).

Data generated in the first replication stage were then meta-analysed with those from the Austrian CORSA and Czech CCS studies, including a total of 15,076 subjects (8006 CRC cases and 7070 controls). Importantly, the meta-analysis of all study cohorts confirmed that carriers of the *DAPK2*_rs11631973G_ allele had a significantly increased risk of developing CRC (OR_Meta_ = 1.13, 95%CI 1.07–1.19, *p* = 0.000022, *p*_Corrected_ = 0.0041; Table 2 and Table S7). It is worth noting that the meta-analysis of all study cohorts also revealed a potentially interesting association of the *ATG5*_rs546456_ SNP in modulating the risk of developing the disease. Each copy of the *ATG5*_rs546456T_ allele additively increased the risk of developing the disease by 8% (OR_Meta_ = 1.08, 95%CI 1.04–1.14, *p* = 0.00062; Table 2 and Table S7). The associations with the *DAPK2* and *ATG5* SNPs did not show any population heterogeneity.

**Figure 2:**
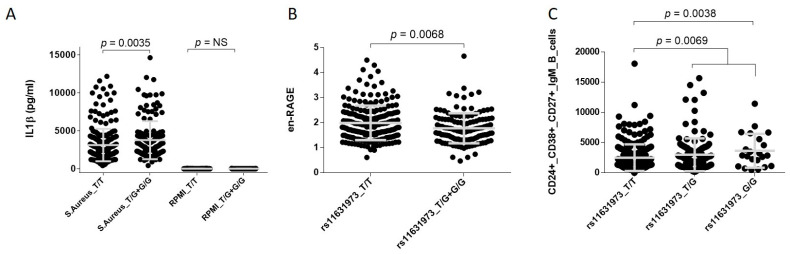
Functional characterisation of the *DAPK2* variant. NS, not significant. (**A**) PBMCs were stimulated with *S. Aureus.* (**B**) en-RAGE levels were measured in serum of 343 healthy subjects. (**C**) Percentage of CD24+CD38+CD27+IgM+ transient B cells were quantified considering CD3-CD19+ B cells as grandparent cell population (cell populations from which this population was isolated). CD3-CD19+ B cells were used to normalize the analysis in order to avoid inter-experimental bias and to increase statistical power. Results remain very similar when CD24+CD38+ B cells were used as parent cell population for normalization (*p* = 0.0073).

Mechanistically, we found that carriers of the *DAPK2*_rs11631973G_ showed increased levels of IL1 β after stimulation of PBMCs with *Staphylococcus aureus* (*p* = 0.0035; Figure 2A) and lower levels of serum en-RAGE (*p* = 0.0068; Figure 2B), a protein that hampers the spread and virulence of *Helicobacter pylori*.

In addition, we found that subjects harbouring the *DAPK2*_rs11631973G_ allele showed slightly increased levels of CD24 + CD38 + CD27 + IgM + B cells (*p* = 0.0038; Figure 2C), a subset of cells enriched in CRC patients. Although none of the functional data remained significant after multiple testing, these results together with those reporting a correlation between *DAPK2* SNPs and *DAPK2* mRNA expression in multiple tissues, including oesophagus/oesophageal junction and oesophagus/muscularis (*p*-values ranging from 7.6 × 10^−6^ to 2.3 × 10^−4^; Table S7), pointed to a role of the *DAPK2* locus in modulating CRC risk likely through the regulation of host immune responses against components of the human microbiota.

**Figure 3:**
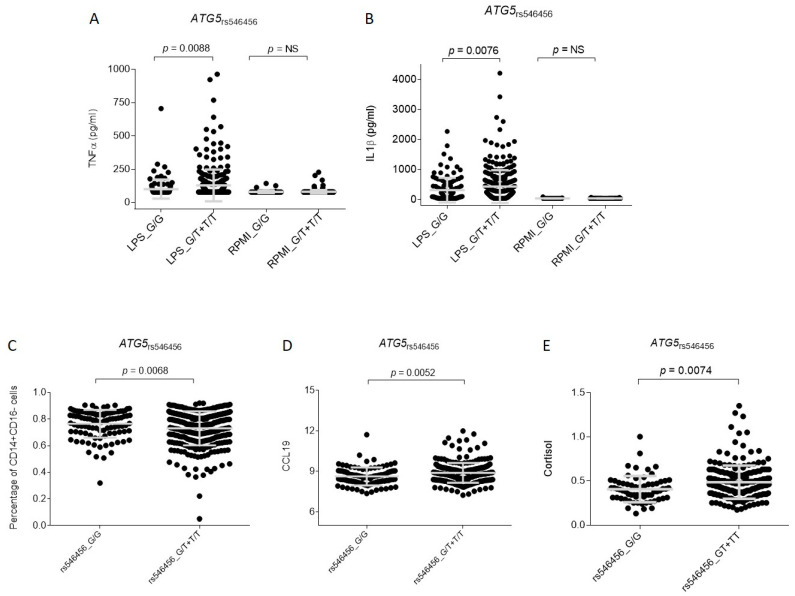
Functional characterisation of the *ATG5*_rs546456_ variant. NS, not significant. (**A**,**B**) PBMCs were stimulated with LPS. (**C**) Percentages of CD14+CD16− cells were quantified considering monocytes CD14+ as the grandparent cell population. (**D**) Serum levels of CCL19 and (**E**) cortisol were measured in the sera of 343 healthy subjects.

On the other hand, in support of a functional role of the *ATG5*_rs546456_ SNP in modulating disease risk, we found that, after the stimulation of PBMCs with LPS, carriers of the *ATG5*_rs546456T_ allele had increased levels of TNF α and IL1 β (*p* = 0.0088 and *p* = 0.0076, respectively; Figure 3A,B). In addition, we found that carriers of the *ATG5*_rs546456T_ allele tended to have decreased levels of classical monocytes in blood (CD14 + CD16−; *p* = 0.0068; Figure 3C) and increased levels of serum CCL19 and cortisol (*p* = 0.0052 and *p* = 0.0074; Figure 3D,E). No association between *ATG5*_rs546456_ SNP and autophagy flux was detected (Table S8 and Figure S1). Again, although none of the functional results could be considered statistically significant after correction for multiple testing, these results together with those from the GTex portal demonstrating a correlation of this marker with *ATG5* mRNA expression in muscle skeletal tissue (*p* = 2.7 × 10^−9^) suggested a weak but still functional role of the *ATG5* locus in the pathogenesis of CRC at multiple levels.

Finally, it is also important to mention that the associations of the *NRG3*, *TP73* and *EGFR* SNPs with the risk of developing CRC in the DACHS and CRCGen cohorts could not be confirmed either in the CORSA and/or Czech CCS cohorts, which dismissed the idea of a relevant biological role of these loci on the risk of developing CRC (Table S7). 

## 4. Discussion

This comprehensive study reports, for the first time, the association of autophagy-related genes with CRC risk. In the meta-analysis of four European cohorts with a total of 8006 CRC cases and 7070 controls, *DAPK2* and *ATG5* loci were associated with a risk of CRC. Functional characterisation of the SNPs showing the strongest associations revealed no association with autophagy flux, but a microbiome-immunity link in genetically susceptible individuals that might lead to CRC development. 

The strongest association was found for the *DAPK2*_rs11631973_ polymorphism within the *DAPK2* gene. Each copy of the *DAPK2*_rs11631973G_ allele increased the risk of developing CRC by 13%. *DAPK2* encodes death-associated protein kinase 2 that belongs to a family of proapoptotic Ca^2+^/calmodulin-regulated serine/threonine kinases. Although it is thought to be a tumour suppressor in haematological malignancies [32,33], DAPK2, in contrast to other DAPK family proteins, has not been identified as a tumour suppressor in solid tumours. However, in support of its possible role in colorectal tumorigenesis, it has been demonstrated that DAPK2 is involved in the regulation of haematopoiesis, cellular motility [34], and neutrophil differentiation [35]. In addition, inactivation of the *DAPK2* gene has been associated with cancer development [36,37]. These studies, along with more recent studies using DAPK2 inhibitors, have opened a new window for cancer treatment. However, data related to the role of this gene in determining CRC risk are sparse. In this regard, our functional experiments showed that PBMCs from carriers of the *DAPK2*_rs11631973G_ allele had increased levels of IL1 β after stimulation with *Staphylococcus aureus*, which led to the hypothesis that the *DAPK2* locus, known to be involved in modulating neutrophil and eosinophil function, might influence CRC risk through the upregulation of IL1 β production by granulocytes in response to components of the intestinal microbiota and, thereby, promote chronic inflammation. These findings are in line with those reporting that high levels of neutrophil-derived IL1 β alter the colonic epithelial barrier [38], induces tumorigenesis, and correlates with poor prognosis in solid tumour patients [39]. Likewise, in support of the hypothesis suggesting a role of the *DAPK2*_rs11631973_ SNP in modulating granulocyte function, we also found that carriers of the *DAPK2*_rs11631973G_ allele showed decreased serological levels of en-RAGE, a protein encoded by the *S100A12* gene and secreted by granulocytes that have an inhibitory effect on the spread and virulence of *Helicobacter pylori*. This result suggested that the *DAPK2*_rs11631973_ SNP might also have an impact on CRC risk by determining the immune response against *H. pylori* infection, a pathogen consistently associated with CRC development [40] among other cancers [41]. Interestingly, we also found that subjects harbouring the *DAPK2*_rs11631973G_ allele showed slightly increased levels of CD24 + CD38 + CD27 + IgM + B cells, a subset of transitional B cells frequently found in leukocytes from CRC patients that regulate T cell-mediated proinflammatory responses and correlate with advanced disease stages [42]. Furthermore, in silico data from Haploreg showed that this variant correlates with enhanced promoter activity exclusively in primary neutrophils and that it is located among H3K4me1 histone marks in the rectal mucosa. Data from GTex portal also showed that the *DAPK2*_rs11631973G_ allele correlates with DAPK2 mRNA expression levels in oesophagus/gastroesophageal junction and oesophagus/muscularis, among other tissues. Although none of the functional results remained significant after correction for multiple testing, altogether these results pointed to a role of the *DAPK2* locus in CRC pathogenesis through host immune responses.

Another interesting finding was the association of the *ATG5* SNP with CRC risk that remained only marginally significant after multiple testing corrections. In line with our genetic data, functional experiments suggested a role of this locus in the modulation of CRC risk. ATG5 (autophagy related gene 5) encodes for a 275 amino acid protein involved in the control of autophagic vesicle formation but also in the mitochondrial response to oxidative damage, T cell differentiation, and immune responses to microorganisms. Although the role of *ATG5* in CRC remains unclear due to the controversial results between in vitro and in vivo studies, it has been reported that the *ATG5* locus was lost in more than 20% of CRC patients and that heterozygous or complete deletion of *ATG5* led to increased cellular death and tumour burden and enhanced antitumor efficacy of IFN γ [43]. Mechanistically, it has been reported that heterozygous deletion of *ATG5* activated *EGFR* and Wnt/β–catenin pathways in adenomas of Apc(Min/+) mice leading to the enhancement of the IFN γ-dependent inhibition of these pathways [43]. Moreover, more recent studies have suggested that the controversial role of ATG5 in CRC might be due to the compensatory activation of autophagy-related proteins (AKT, RICTOR and mTOR) in response to autophagy inhibition [44], which have also been associated with CRC prognosis [45]. In contrast to the notion of a role of the ATG5 locus in modulating autophagy, our study has suggested that the *ATG5*_rs546456_ SNP might influence CRC risk by modulating host immune responses. We observed that carriers of the *ATG5*_rs546456T_ allele showed increased levels of TNF α and IL1 β after the stimulation of PBMCs with LPS and tended to have decreased levels of classical monocytes and increased levels of serum CCL19. These functional results were also in line with those suggesting *ATG5* in the modulation of neutrophil-derived IL1 β levels in response to LPS [46], but also in the regulation of classical monocytes in blood and serum levels of CCL19, a relevant chemokine that play a key role in the control of CRC cell proliferation, migration and angiogenesis [47]. In line with this notion, previous studies have demonstrated that LPS stimulates the non-canonical inflammasome to induce the production of IL1 β in neutrophils but also other myeloid cells including macrophages, and that this effect on the inflammasome is mediated, at least in part, by ATG5 [48]. Furthermore, it has been demonstrated that autophagy inhibits neutrophil apoptosis and that the siRNA-mediated silencing of ATG5 resulted in accelerated spontaneous apoptosis but attenuated TNF α-induced apoptosis, which suggested a context-specific effect of ATG5 on immune cell survival [49]. Interestingly, we also found a weak correlation between the *ATG5*_rs546456T_ allele and increased levels of cortisol in serum, which was in agreement with previous studies suggesting that cortisol is associated with immune deregulation, cancer development, disease progression [50] and a more aggressive metastasis [51]. Previous studies have also demonstrated that cortisol, acting synergistically with catecholamines, may facilitate cancer cell growth and potentiate the release of TNF α and IL1 β. Even though neither the genetic association of the *ATG5*_rs546456_ SNP with CRC risk nor functional data remained significant after correction for multiple testing, altogether these results suggest a role of the *ATG5* locus in modulating immune cells (probably neutrophils and macrophages) and their function in activating tumorigenic pathways in CRC. 

Finally, it is important to mention that this study has both strengths and drawbacks. The major strengths of our study are the comprehensive analysis of inherited genetic variation in 234 autophagy-related genes reported in the autophagy database (http://autophagy.lu/index.html, accessed on 13 December 2019) and the inclusion of four large European populations including a total of 15,076 subjects, 8006 CRC cases, and 7070 controls. In the meta-analysis including all study cohorts, we had 80% power to detect an odds ratio of 1.12 (α = 0.00027) for an SNP with a frequency of 0.25, which emphasised the feasibility of the study design. Likewise, we comprehensively analysed the impact of autophagy-related SNPs in modulating blood cell counts, steroid hormones, serum and plasma metabolites, and immune responses in a large cohort of healthy subjects. Another important strength of this study was the experimental analysis assessing the effect of autophagy SNPs in modulating the autophagy flux in PBMCs left untreated or treated with metformin or bafilomycin. An important drawback of this study was its multicentric nature that placed inevitable limitations such as the impossibility of uniformly collect mutation profiles (including *KRAS*^G12^ but also *APC*, *TP53*, *EGFR*, *BRAF*, *LOH*, *PIK3CA* and *TGFBR*) for a significant set of patients.

## 5. Conclusions

This study reports, for the first time, a functional impact of *DAPK2* and *ATG5* loci in modulating CRC risk and provides new insights into the functional role of *DAPK2* and *ATG5* polymorphisms in disease pathogenesis.

## Figures and Tables

**Table 1:** Meta-analysis of the association between autophagy-related variants and CRC risk in the DACHS and CRCGen populations.

Gene	Variant_dbSNP	Risk Allele	DACHS Cohort (*n* = 7998)		CRCGen Cohort (*n* = 2024)		Meta-Analysis (*n* = 10,022)		*p_Het_*
			OR (95% CI) ^a^	*p*-Value	OR (95% CI) ^a^	*p*-Value	OR (95% CI) ^a^	*p*-Value	
*NRG3*	rs11196336	C	1.18 (1.10–1.28)	0.00002	1.09 (0.93–1.28)	0.3125	**1.17 (1.09–1.25)**	**0.0000185**	**0.354**
*DAPK2*	rs11631973	G	1.15 (1.06–1.24)	0.00060	1.17 (1.00–1.36)	0.0563	**1.15 (1.07–1.23)**	**0.0000799**	**0.846**
*EGFR*	rs2075108	A	1.15 (1.03–1.28)	0.01308	1.39 (1.11–1.73)	0.0035	1.19 (1.08–1.31)	0.000457	0.134
*TP73*	rs4648553	A	1.10 (1.02–1.18)	0.01103	1.21 (1.05–1.39)	0.0104	1.12 (1.05–1.20)	0.000590	0.254
*LOC100128105*	rs6565506	C	1.09 (1.01–1.17)	0.02129	1.21 (1.04–1.41)	0.0122	1.11 (1.04–1.19)	0.001597	0.214
*ATG5*	rs546456	T	1.09 (1.03–1.16)	0.00574	1.10 (0.97–1.25)	0.1324	1.09 (1.03–1.16)	0.001700	0.918

Abbreviations: SNP, single nucleotide polymorphism; OR, odds ratio; CI, confidence interval. Estimates were adjusted for age, sex, and principal components. Significant results are in bold after correction for multiple testing considering a significant threshold of 0.00027. ^a^ Association estimates were calculated according to a log-additive model of inheritance.

**Table 2:** Association estimates of the most interesting autophagy-related variants and CRC risk in the four study cohorts.

Gene_Variant	Risk Allele	DACHS Cohort (*n* = 7998)		CRCGen Cohort (*n* = 2024)		CORSA (*n* = 1816)		Czech Republic CCS (*n* = 3238)		OR (95% CI) ^a^	*p*-Value	*p_Corr_*	*p_Het_*
		OR (95% CI) ^a^	*p*-Value	OR (95% CI) ^a^	*p*-Value	OR (95% CI) ^a^	*p*-Value	OR (95% CI) ^a^	*p*-Value				
*DAPK2*_rs11631973_	G	1.15 (1.06–1.24)	0.0006	1.17 (1.00–1.36)	0.056	1.12 (0.96–1.30)	0.14	1.07 (0.96–1.20)	0.25	**1.13 (1.07–1.19)**	**2.19 × 10^−5^**	**0.0041**	0.760
*DAPK2*_rs11635284_	G	1.13 (1.05–1.22)	0.001	1.19 (1.02–1.39)	0.032	1.12 (0.96–1.30)	0.15	1.06 (0.95–1.19)	0.28	**1.12 (1.06–1.18)**	**4.77 × 10^−5^**	**0.0087**	0.679
*ATG5*_rs546456_	T	1.09 (1.03–1.16)	0.006	1.10 (0.97–1.25)	0.132	1.07 (0.94–1.22)	0.33	1.06 (0.95–1.18)	0.28	**1.08 (1.04–1.14)**	**0.00062**	0.11	0.958
*ATG5*_rs490010_	G	1.08 (1.01–1.15)	0.020	1.09 (0.96–1.23)	0.181	1.09 (0.96–1.25)	0.18	1.06 (0.95–1.19)	0.30	**1.08 (1.03–1.13)**	**0.002**	NS	0.985
*NRG3*_rs11196336_	C	1.18 (1.10–1.28)	0.00002	1.09 (0.93–1.28)	0.313	1.11 (0.95–1.30)	0.19	0.94 (0.82–1.08)	0.25	**1.11 (1.05–1.18)**	**0.00025**	**0.045**	**0.041**
*EGFR*_rs2075108_	A	1.15 (1.03–1.28)	0.013	1.39 (1.11–1.73)	0.0035	0.86 (0.68–1.08)	0.20	0.89 (0.70–1.14)	0.36	**1.10 (1.01–1.20)**	**0.025**	NS	**0.007**
*TP73*_rs4648553_	A	1.10 (1.02–1.18)	0.011	1.21 (1.05–1.39)	0.010	0.87 (0.75–1.01)	0.075	1.11 (0.96–1.28) *	0.17	**1.08 (1.02–1.14)**	**0.0039**	NS	**0.013**

Abbreviations: SNP, single nucleotide polymorphism; OR, odds ratio; CI, confidence interval; NS, not significant. Estimates were adjusted for age, sex, and principal components. Significant results are in bold after correction for multiple testing considering a significant threshold of 0.00027. ^a^ Association estimates were calculated according to a log-additive model of inheritance. * Values based on results from 1068 CRC cases and 850 healthy controls.

## Data Availability

The genotype data used in the present study are available from the corresponding authors upon reasonable request. Functional data used in this project have been meticulously catalogued and archived in the BBMRI-NL data infrastructure (https://hfgp.bbmri.nl/, accessed on 13 December 2019) using the MOLGENIS open-source platform for scientific data [52]. This allows flexible data querying and download, including sufficiently rich metadata and interfaces for machine processing (R statistics, REST API) and using FAIR principles to optimise findability, accessibility, interoperability and reusability [53,54].

## Supplementary Materials

The following are available online at https://www.mdpi.com/2072-6694/13/6/1258/s1, Figure S1: Representative Western blot plot of the autophagy analysis, Table S1: Baseline characteristics of the DACHS population, Table S2: Baseline characteristics of the CRCGen population, Table S3: List of selected genes, Table S4: Genome-wide genotyping platforms used to genetically characterise the DACHS cohort, Table S5: Cell types analysed either in whole blood or peripheral mononuclear blood cells, Table S6: Serum and plasma metabolites measured in the HFGP cohort, Table S7: Meta-analysis of all study cohorts, Table S8: Correlation between DAPK2 and ATG5 SNPs and autophagy flux.
