# Supplementary material for: Polymorphisms within Autophagy-Related Genes Influence the Risk of Developing Colorectal Cancer: A Meta-Analysis of Four Large Cohorts
Source: Cancers (Basel). 2021 Mar 12;13(6):1258. doi: 10.3390/cancers13061258 (PMC7998818; doi:10.3390/cancers13061258)
Supplement: Supplementary file 1 [file cancers-13-01258-s001.zip › supply/Supplementary Figure.docx]

Supplementary Materials

Polymorphisms within Autophagy-Related Genes Influence the Risk of Developing Colorectal Cancer: A Meta-Analysis of Four Large Cohorts

Juan Sainz, Francisco José García-Verdejo, Manuel Martínez-Bueno, Abhishek Kumar,
José Manuel Sánchez-Maldonado, Anna Díez-Villanueva, Ludmila Vodičková,
Veronika Vymetálková, Vicente Martin Sánchez, Miguel Inacio Da Silva Filho,
Belém Sampaio-Marques, Stefanie Brezina, Katja Butterbach, Rob ter Horst, Michael Hoffmeister, Paula Ludovico, Manuel Jurado, Yang Li, Pedro Sánchez-Rovira, Mihai G. Netea, Andrea Gsur, Pavel Vodička, Víctor Moreno, Kari Hemminki, Hermann Brenner, Jenny Chang-Claude
and Asta Försti


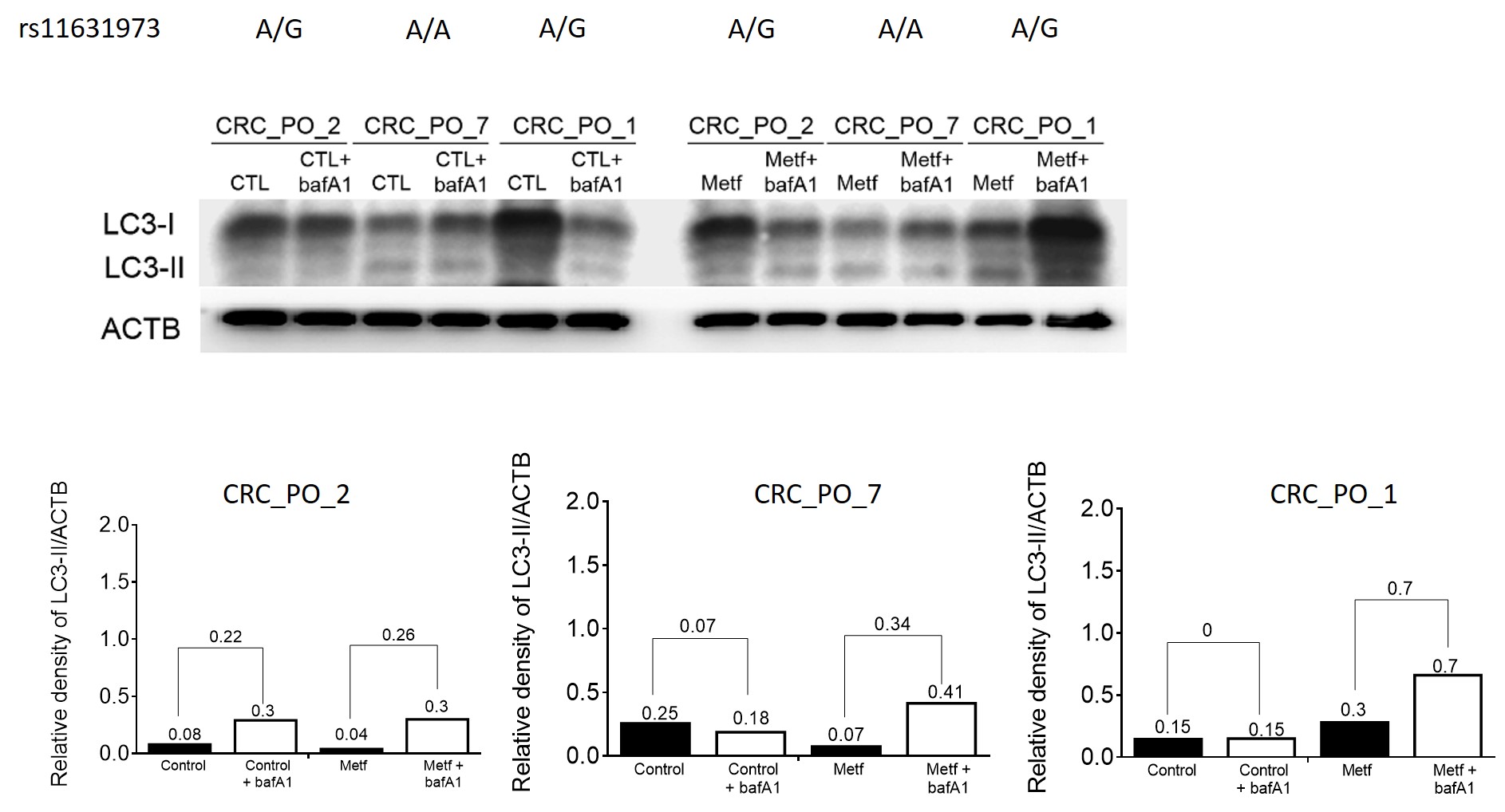


**Figure S1.** Representative Western blot plot of the autophagy analysis.

| 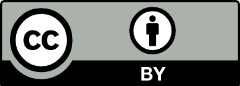 | © 2021 by the authors. Licensee MDPI, Basel, Switzerland. This article is an open access article distributed under the terms and conditions of the Creative Commons Attribution (CC BY) license (http://creativecommons.org/licenses/by/4.0/). |
| --- | --- |
